# Ambient Pressure Chemical Vapor Deposition of Flat and Vertically Aligned MoS_2_ Nanosheets

**DOI:** 10.3390/nano12060973

**Published:** 2022-03-16

**Authors:** Pinaka Pani Tummala, Christian Martella, Alessandro Molle, Alessio Lamperti

**Affiliations:** 1 Institute for Microelectronics and Microsystems (CNR-IMM), Unit of Agrate Brianza, via C. Olivetti 2, I-20864 Agrate Brianza, Italy; pinakapani.tummala@mdm.imm.cnr.it (P.P.T.); christian.martella@mdm.imm.cnr.it (C.M.); alessandro.molle@mdm.imm.cnr.it (A.M.); 2 Dipartimento di Matematica e Fisica, Università Cattolica del Sacro Cuore, via della Garzetta 48, I-25133 Brescia, Italy; 3 Department of Physics and Astronomy, Katholieke Universiteit Leuven (KU Leuven), Celestijnenlaan 200D, 3001 Leuven, Belgium

**Keywords:** MoS_2_, 2D TMD, AP-CVD, large area growth, growth selectivity, pattern substrates

## Abstract

Molybdenum disulfide (MoS_2_) got tremendous attention due to its atomically thin body, rich physics, and high carrier mobility. The controlled synthesis of large area and high crystalline monolayer MoS_2_ nanosheets on diverse substrates remains a challenge for potential practical applications. Synthesizing different structured MoS_2_ nanosheets with horizontal and vertical orientations with respect to the substrate surface would bring a configurational versatility with benefit for numerous applications, including nanoelectronics, optoelectronics, and energy technologies. Among the proposed methods, ambient pressure chemical vapor deposition (AP-CVD) is a promising way for developing large-scale MoS_2_ nanosheets because of its high flexibility and facile approach. Here, we show an effective way for synthesizing large-scale horizontally and vertically aligned MoS_2_ on different substrates such as flat SiO_2_/Si, pre-patterned SiO_2_ and conductive substrates (TaN) benefit various direct TMDs production. In particular, we show precise control of CVD optimization for yielding high-quality MoS_2_ layers by changing growth zone configuration and the process steps. We demonstrated that the influence of configuration variability by local changes of the S to MoO_3_ precursor positions in the growth zones inside the CVD reactor is a key factor that results in differently oriented MoS_2_ formation. Finally, we show the layer quality and physical properties of as-grown MoS_2_ by means of different characterizations: Raman spectroscopy, scanning electron microscopy (SEM), photoluminescence (PL) and X-ray photoelectron spectroscopy (XPS). These experimental findings provide a strong pathway for conformally recasting AP-CVD grown MoS_2_ in many different configurations (i.e., substrate variability) or motifs (i.e., vertical or planar alignment) with potential for flexible electronics, optoelectronics, memories to energy storage devices.

## 1. Introduction

Over the last decade, transition metal dichalcogenides (TMDs) have attracted immense interest because of their outstanding electrical, optical and chemical properties [1,2,3]. These TMDs are typically attributed as MX_2_, where M is a transition metal atom (i.e., molybdenum, Mo) and X refers to a chalcogen atom (i.e., sulfur, S) to form a TMD (i.e., molybdenum disulfide, MoS_2_). Monolayer MoS_2_, with a direct band gap of 1.8 eV and a three atoms thick nanosheet, shows potential applications in the fields of electronics, optoelectronics and valleytronics due to optical transparency, high carrier mobility, no-dangling bonds and atomically thickness [3,4,5]. In addition, conformal growth of MoS_2_ nanosheets over pre-patterned substrates enables an added advantage for effective routes towards fabrication; the miniaturization of integrated circuits and structurally excellent TMD materials are essential for flexible optoelectronics such as LEDs and may lead to bandgap and exciton engineering through local strain generation due to excellent conformal growth and layer bendability [6,7]. The growth on the patterned substrate is also fundamental for the exploitation of the anisotropy by design in MoS_2_ and related TMDs, thus enabling the engineering of the electronic band structure as a function of the local strain. Anisotropic effects are relevant in a broad range of applications. For instance, when we consider the resulting nanosheets as 2D rippled membranes, we are devising new physical concepts to enhance the optical, plasmonic, and catalytic performance of the pristine materials. Further, direct growth on engineered patterned substrates promotes strain engineering, with implications on relevant properties of TMDs such as the thermal/electronic transport or the exciton physics, which are dramatically affected by the anisotropy-dependent degree of strain [8,9]. On another front, direct growth of high crystalline MoS_2_ on conductive substrates would open up a new pathway for easy integration in memory devices by serving as a bottom electrode and also providing an underlying conductive substrate [10]. MoS_2_ and other TMDs, such as TaS_2_, are considered as diffusion barriers in ultra-scaled microelectronics (<5 nm technology node) directly in contact with TaN or Cu, which are commonly used as interconnects in back-end-of-line compatible processes [11,12]. In this regard, a significant demand to develop an efficient approach to direct growth of MoS_2_ on metal or conductive substrates at a large scale is still demanding, although the high process temperature poses severe constraints in terms of substrate stability during growth. In contrast, multilayered MoS_2_ with an indirect band gap of 1.3 eV, disordered structures with exposed edge defects, chemical stability and high surface area are a favorable ground for heterogeneous catalysis reaction with promising impact on hydrogen storage and fuel cells [4,13,14,15]. It is known that excellent catalytic activities of MoS_2_ are greatly enhanced by using conductive layers as a growth substrate, thus providing cost-effective, high-performance catalysts in electrocatalysis over Pt [13].

Before the practical applications, so far, several considerable efforts have been focused on the preparation of large-scale MoS_2_. Approaches, such as physical and chemical exfoliation, chemical synthesis, atomic layer deposition, laser annealing, physical vapor deposition and chemical vapor deposition, have been reported [16,17,18,19]. Among the proposed methods, chemical and mechanical exfoliation, physical vapor deposition, and chemical vapor deposition schemes are the most used ones [18,20]. Specifically, chemical exfoliation or sonication are versatile methods for the low cost, scalable production of monolayer 2D materials [16,21]. Mechanical, or tape, exfoliation allows to obtain high crystal quality of monolayer MoS_2_ but is beneficial for fundamental property studies only [17]. Indeed, small size, nonuniform thickness and agglomeration in solution are drawbacks of this method. An explicit understanding of these variables is critical for precise control of MoS_2_ morphology with large coverage during their growth. Such desired knowledge could further allow for the synthesis of other TMDs that consists of both vertical and horizontally grown layer structures [22].

Ambient pressure chemical vapor deposition (AP-CVD) is a facile, efficient, scalable method to grow large-scale monolayer MoS_2_ aiming for the fabrication of integrated devices [23,24]. Conversely, CVD is also a flexible, cost-effective, and scalable process for growth optimization from flat horizontal to vertically oriented structured MoS_2_ for energy storage applications. Despite atomically thin MoS_2_ have been successfully grown on SiO_2_/Si, also by our research team, [24] there are still some limitations in extending this process to direct synthesis of large-area monolayers on different substrates such as pre-patterned and metal or conductive substrates. In this respect, computational studies, based on ab-initio methods, such as density functional theory, provided useful insights in predicting and clarifying the growth mechanism involved in CVD growth of TMDs and similar systems [25,26,27]. Having the application target in mind, precise tuning of the growth orientations of horizontally and vertically aligned MoS_2_ is critically important to benefit from their tailored materials properties and device functionalities in various fields. Furthermore, by modifying the configurational design inside the CVD reactor, the direct growth of large-scale MoS_2_ on any arbitrary conductive substrate would open up an easy route for bottom contacts, thus facilitating MoS_2_ integration in devices.

In this work, we investigated the growth behavior of mono to a few layers of MoS_2_ from molybdenum trioxide (MoO_3_) and sulfur (S) solid powders as a precursor in the AP-CVD process. We explicitly demonstrated the local changes of the S to MoO_3_ precursor positions in the growth zone inside the CVD reactor, which play a key factor in the changing of MoS_2_ nanosheets orientation. We successfully synthesized high-quality MoS_2_ flat monolayers and vertically aligned bulk MoS_2_ up to cm^2^ scale with good uniformity on different substrates such as SiO_2_/Si, pre-patterned SiO_2_ and TaN. In addition, we grew isolated single domains to continuous MoS_2_ conformally on the pre-patterned surface without any ruptures. The growth formation, crystallinity, extension of monolayer and vertically grown MoS_2_ layers were characterized by a series of techniques such as Raman spectroscopy, X-ray photoelectron spectroscopy (XPS), scanning electron microscopy (SEM) and photoluminescence (PL). We conclude with an outlook on the prospective scientific future of device developments based on MoS_2_ grown by optimized CVD methods.

## 2. Materials and Methods

**Figure 1:**
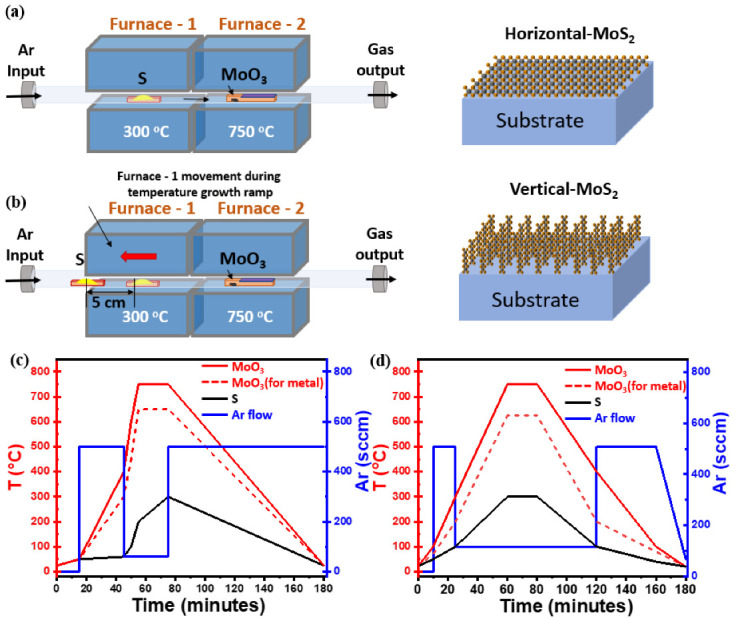
(**a**) Schematic diagram of the horizontal two-zone CVD furnace for the synthesis of flat-MoS_2_. (**b**) Modified CVD setup used to synthesize vertical-MoS_2_ nanosheets by adjusting the boat position during the growth temperature ramp on different substrates (**c**) Different steps of temperature profile (left *y*-axis) adopted for the synthesis of flat-MoS_2_ nanosheets with growth ramp at 750 °C (solid line, SiO_2_/Si) and 650 °C (dash line, TaN) for 20 min; in the same graph the Ar flux changes during the CVD process are also plotted (blue solid line, right *y*-axis). (**d**) Three step temperature profile (left *y*-axis) used for the synthesis of vertical-MoS_2_ nanosheets with growth ramp at 750 °C (solid line, SiO_2_/Si) and 625 °C (dash line, TaN) for 20 min; in the same graph the Ar flux changes during the CVD process are also plotted (blue solid line, right *y*-axis).

MoS_2_ nanosheets reported in this work has been grown using atmospheric-pressure chemical vapor deposition (AP-CVD), in which powder of sulfur (S, 99.98%, Sigma-Aldrich, Darmstadt, Germany) and molybdenum trioxide (MoO_3_, 99.97%, Sigma-Aldrich, Darmstadt, Germany) were used as precursor sources. Amorphous SiO_2_(50 nm)/Si (100) substrates were utilized for initial MoS_2_ growth experiments. Furthermore, we extended our optimized growth recipe to pre-patterned SiO_2_ and tantalum nitride (TaN) substrates. The growth procedure takes place inside a two-zone-furnace AP-CVD apparatus with a 2” quartz tube with a length of 150 cm where upstream (S) and downstream (MoO_3_) containing boats are precisely positioned, as shown in Figure 1a. For the synthesis of horizontal and vertical aligned films, a similar setup was used but with some changes, as detailed in individual sections below.

### Synthesis of Horizontally Aligned MoS_2_ Nanosheets

During the CVD, S and MoO_3_ powders with the amount of 200 mg and 1 mg, respectively, were contained in crucibles and placed near to heating zones of upstream and downstream furnaces. A SiO_2_(50 nm)/Si substrate previously cleaned was kept face-down above the MoO_3_ containing crucible. Substrate cleaning was performed with acetone and isopropanol followed by immersion in piranha solution of composition H_2_O_2_:H_2_SO_4_ (1:3) for 2 h to make the surface hydrophilic, rinsed with deionized water and finally N_2_ blown to dry. Following the previous steps, the substrate was conditioned with PTAS (Perylene-3,4,9,10-tetracarboxylic acid tetrapotassium salt) seeding promotor, 0.29 mg mixed in 10 mL distilled water solution, dropped on the clean SiO_2_/Si substrate using micropipette, then spun at 600 rpm for 60 s. The use of PTAS molecules as a seed promotor during the process of substrate conditioning has been demonstrated to improve the size of MoS_2_ nanodomains and the overall MoS_2_ nanosheets synthesis by promoting lateral flat growth at the expense of the “3D-like” vertical growth [24,28]. In other words, without PTAS, we could not reach satisfactory surface coverage; the 2D-like MoS_2_ domains are small in size, less than 10 μm, and they are isolated, leaving a relevant region of the exposed substrate surface with no MoS_2_ growth, as shown in representative SEM image in Figure S1 in Supplementary Materials (SM). The S and MoO_3_ containing boats are placed in the middle of each heat zone of the furnace, as shown in the schematic in Figure 1a. The temperature profile during the 2 h long CVD process reaches a maximum of 300 °C for sulfur and 750 °C for MoO_3_ during the effective growth step of 20 min; concomitantly, Ar flux varies from 60 sccm, during the 20 min growth time, to 500 sccm in the other different steps during the CVD process, as shown in Figure 1c. The temperature profile for S, with a maximum temperature of 300 °C, grants the control of the sulfur sublimation rate. For the MoO_3_-containing furnace, 750 °C is selected to facilitate the chemical reactivity of Mo at the sample surface while avoiding the formation of MoS_x_O_y_ by-products. In the case of TaN substrates, we reduced the growth temperature to 650 °C in the MoO_3_ heating zone to preserve TaN from evaporation, as shown in Figure 1c (dashed line). The temperature profile for S was kept identical to the case of SiO_2_/Si substrate, while the Ar flux varied from 100 sccm during the effective growth step up to 600 sccm in the other steps.

### Synthesis of Vertically Aligned MoS_2_ Nanosheets

In the case of vertically aligned MoS_2_, we used S and MoO_3_ powders in the amount of 300 mg and 1 mg, respectively, as we modified the apparatus setup according to the growth conditions. Here it is worth noting that, during the sample conditioning, we do not make use of any seeding promotor. Initially, we positioned the sulfur source at the beginning of the heat zone, far from (downstream) furnace 1, to prevent any pre-evaporation of sulfur, as shown in Figure 1b. The MoO_3_ source, along with the downface substrate, is placed at the centre of the heat zone in the (upstream) furnace 2. Once the MoO_3_ containing furnace reaches the maximum of the ramping up temperature, namely 750 °C, we slowly introduce the sulfur vapors by moving the S furnace 1 (S temperature zone) manually towards the molybdenum direction and releasing the Ar carrier gas of 100 sccm. In this growth method, the carrier gas flow is kept low and constant for a long time during some CVD process steps. The delayed introduction of the sulfur source together with its high excess at the position where the chemical reaction between the MoO_3_ and S happens creates a concentration gradient normal to the substrate surface, promoting the out-of-plane vertical growth of MoS_2_ possibly due to the Mullins–Sekerka mechanism, thus ultimately leading to vertical oriented MoS_2_ growth, as mentioned in the literature [22,29]. Here, the delayed reaction takes place in vapor phase between the S and MoO_3_. In addition, the absence of seed promotors during the pre-treatment of the substrate surface changes the surface wettability, which is also to be considered a key factor for vertical growth. Figure 1d shows the three steps temperature profile, together with the Ar flux profile, followed during the vertically aligned MoS_2_ growth. The process starts with the introduction of the source precursors, followed by temperature ramp steps and natural cool down of the apparatus. Differently from the horizontal growth case, here we reduced the growth temperature to 625 °C for TaN substrate to avoid any possible substrate reactivity as indicated by the red dash line in Figure 1d.

### Characterization Methods

The morphology of the as-grown samples was first examined by scanning electron microscopy (SEM) using a Zeiss-SUPRA 40 field-emission SEM device (Oberkochen, Germany) in bright field mode. Photoluminescence (PL) and confocal micro-Raman spectroscopy were performed using a Renishaw In-Via spectrometer (New Mills, Kingswood, Wotton-under-Edge, UK) equipped with a solid-state laser source of excitation wavelength 514 nm (2.41 eV) in backscattering configuration. Particular care was put in the laser power, reduced to 5% of the nominal power (i.e., below 1 mW) to avoid sample damage. Complementary PL was acquired with the same Raman equipment by changing the instrument configuration. X-ray photoelectron spectroscopy (XPS) measurements were acquired on a PHI 5600 instrument equipped with a monochromatic Al Kα X-ray source with an energy of 1486.6 eV and a concentric hemispherical analyser. The spectra were collected at a take-off angle of 45° and band-pass energy of 58.70 eV. The instrument resolution was 0.5 eV. The spectra were aligned using C 1s (285 eV) as reference.

## 3. Results and Discussion

**Figure 2:**
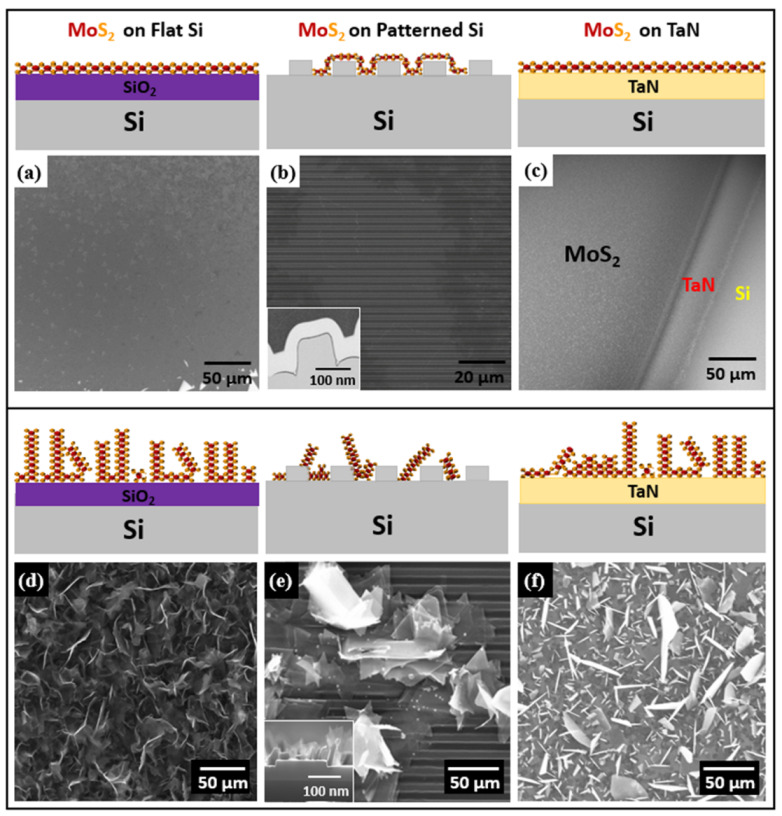
Schematic illustration of horizontal and vertical growth of MoS_2_ using AP-CVD and SEM image (**a**) large area monolayer MoS_2_ grown on flat SiO_2_/Si substrate (**b**) isolated flake of flat monolayer MoS_2_ domain on patterned SiO_2_/Si; inset TEM cross sectional image shows conformal monolayer MoS_2_ growth on patterned trenches (thin dark line). (**c**) flat MoS_2_ on TaN. (**d**–**f**) vertically standing MoS_2_ nanosheets (**d**) on SiO_2_/Si (**e**) on pre-patterned substrate, inset: SEM cross section (**f**) on TaN.

Horizontally and vertically aligned MoS_2_ were synthesized using a two-zone-furnace CVD apparatus on different substrates such as SiO_2_/Si, pre-patterned and conductive substrates. Initial experiments were developed on flat SiO_2_/Si substrates later extended the optimized growth approach to other substrates in terms of flat few layers MoS_2_. Figure 2a–f represents schematics and SEM images of horizontally and vertically aligned MoS_2_ on the different substrates. The as-grown MoS_2_ morphology on flat SiO_2_/Si in the large region is shown by the SEM image as having a lateral size of more than 200 µm of continuous film, as shown in Figure 2a. Here we observed that the triangular MoS_2_ domains merge to yield a large-scale uniform single layer. The MoS_2_ domains are large and tend to connect to each other despite the fact that their orientation on the surface is not controlled. Thus, the amount of grain boundaries and possibly other defects is reduced with respect to the case of relatively small domains randomly oriented in the space. As further evidence, we show large-scale lateral growth of monolayer MoS_2_ up to a centimeter scale in Figures S2 and S3 in Supplementary Materials. Using the same experimental conditions in CVD, we obtained a large-area continuous monolayer MoS_2_ on a patterned SiO_2_/Si substrate (Figure 2b). The cross-sectional view of the monolayer MoS_2_ is also characterized with transmission electron microscopy (TEM), where we observe that the MoS_2_ layer conformally follows the trenches of the pre-patterned substrate as also detailed in the inset TEM cross-sectional image in Figure 2b (see also Figure S4 in Supplementary Materials). By optimizing the CVD growth conditions, we also synthesized highly crystalline few-layers MoS_2_ on TaN. One of the critical parameters during the direct growth of MoS_2_ on the TaN substrate is the growth temperature because of the lower TaN evaporation temperature with respect to the MoS_2_ growth temperature. Although it is well documented that the chemical reaction from Ta(N) and S to form TaS_2_ happens at around 900 °C at ambient pressure [30], we could still note a minimal presence of TaS_2_ phonon modes in Raman measurements, related to the imbalance between the formation of MoS_2_ and TaS_2_ in an atmosphere supersaturated in S, as in our case. Here, we controlled the MoO_3_ temperature by lowering it to 650 °C to preserve TaN film. Figure 2c shows the SEM image of the edge part taken at the interface of MoS_2_ on TaN with exposed silicon on the bottom. The SEM image reveals the domain structure of large-area, horizontally grown monolayer MoS_2_ without any vertically-aligned layers, as shown in Figure 2a–c.

The vertically aligned MoS_2_ growth was obtained by controlling the insertion of sulfur precursor during the temperature ramping-up stage because of substantial differences in the growth kinetics. In CVD growth, we control the sulfur flow that reaches the MoO_3_ source by changing the position of the boat containing sulfur with respect to the heat zone furnace in such a way that the kinetics in the sulfur transport acts as the governing factor for the vertical growth orientation. We speculate that during the growth process, intensive compression between the highly dense bulk MoS_2_ domains leads to the collision with other MoS_2_ islands, thus causing vertical growth. In the vertical growth regime, the sulfur reaction with MoO_3_ yields a concentration gradient normal to the substrate at the substrate surface. This event could have a role in promoting the out-of-plane vertical growth of MoS_2_ due to the Mullins-Sekerka mechanism and significantly reducing the sulfur flow downstream during the growth [22,29]. Figure 2d–f show SEM images of the vertically aligned MoS_2_ nanosheets grown on different substrates. A uniformly covered MoS_2_ grown on flat SiO_2_/Si substrate is clearly shown in Figure 2d. Furthermore, we used the same growth conditions to yield vertical MoS_2_ nanosheets on patterned substrates. Figure 2e clearly shows the presence of triangular vertical MoS_2_ domains after the growth. Bulk MoS_2_ domains appear very bright compared to the few-layers MoS_2_ domains, an indication of a large density of exposed domain edges. In addition, the cross-sectional SEM image in the inset of Figure 2e demonstrates that the as-grown MoS_2_ triangular domains are nearly perpendicular to the patterned substrate surface (see also the cross-section SEM image in Figure S5 in Supplementary Materials). Such findings possibly claim a larger density of defects in the grown MoS_2_ with respect to the flat case. In a simplified picture, in the vertical growth, MoS_2_ domains are formed at a random orientation each other, possibly promoting a high number of defects at their edges, with the final result that a consistent density of defects would be formed. On the other hand, during the CVD growth on TaN, the critical drawback is to control the TaN evaporation temperature and the transition metal source temperature (i.e., molybdenum). Therefore, we precisely investigated different temperatures to preserve the underlying TaN film. Facilitating the same growth approach with the slightly reduced temperature down to 650 °C, we successfully synthesized MoS_2_ on TaN, as shown in Figure 2f, where a top view of vertical MoS_2_/ TaN/SiO_2_ interface is imaged by SEM. Surprisingly, we observed flakes appearing as bright or dark in the SEM image. This fact may be correlated with the competing formation of TaS_2_ flakes along with MoS_2_ as a collateral reaction between S and the TaN substrate according to the observed TaS_2_-related feature in the Raman spectrum in Figure 4d.

**Figure 3:**
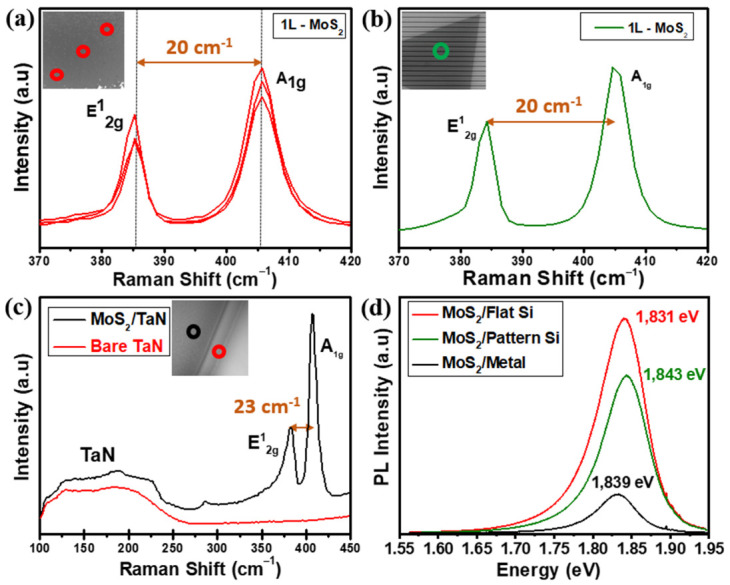
(**a**) Raman spectra obtained at different positions on the as-grown horizontal monolayer MoS_2_ nanosheets on flat SiO_2_/Si; the inset shows the SEM image with the positions of the Raman measures with red open circles (**b**) Raman spectrum taken on monolayer MoS_2_ on pattern substrate; the inset shows the SEM image with the position of the Raman measure with green open circle. (**c**) Raman spectra of bare TaN substrate (red) and after three layers MoS_2_ grown on TaN (black); the inset shows the SEM image with the positions of the Raman measures on TaN substrate with red and MoS_2_ grown on TaN black open circles (**d**) PL spectra of a MoS_2_ on flat Si (red), pattern SiO_2_/Si (green) and TaN (black) substrates.

To gain additional characterization to our MoS_2_ growths, micro-Raman spectroscopy, photoluminescence (PL), and atomic force microscopy (AFM) (Figure S6 in Supplementary Materials) were also employed to probe the structure, optical response and thickness uniformity of flat horizontal MoS_2_ monolayers as shown in Figure 3a–d.

Figure 3a shows the Raman spectra from MoS_2_ nanosheets grown on SiO_2_/Si flat substrate recorded at different positions. The measurements give E^1^_2g_ (in-plane) and A_1g_ (out of plane) phonon modes located at 385.5 and 405.2 cm^−1^, respectively. The wavelength difference of 19.7 cm^−1^ confirms the growth of a MoS_2_ monolayer, consistent with values reported in the literature [31]. Figure 3b shows the Raman measurements on the MoS_2_ grown on the pre-patterned substrate. The measurement gives the same E^1^_2g_ and A_1g_ phonon modes with a frequency difference of ~20 cm^−1^, which validates the growth of a monolayer MoS_2_. As for TaN, we acquired the Raman spectrum of the substrate before and after the MoS_2_ growth, as shown in Figure 3c. The Raman spectrum from bare TaN (red line in Figure 3c) confirms the crystallinity of TaN with a primary first-order acoustic mode (A) centred near 200 cm^−1^ [32,33]. The occurrence of slight variations in the spectra region from 115 cm^−1^ to 230 cm^−1^ in TaN is typically interpreted as stoichiometry modifications, either excess presence of Ta or excess N [32,33]. After MoS_2_ growth (black line in Figure 3c), Raman spectrum evidence the presence of TaN acoustic modes at low Raman shifts together with the presence of MoS_2_ E^1^_2g_ and A_1g_ main phonon modes located at 381.7 cm^−1^ and 404.3 cm^−1^ with a wavelength difference of 22.6 cm^−1^ corresponding to a thickness of 3 layered-MoS_2_ nanosheets. Figure 3d represents the PL spectra obtained on as-grown MoS_2_ on flat SiO_2_/Si (red), patterned SiO_2_/Si (green) and TaN (black) substrate. The PL spectrum shows a high intense PL response peaked around the optical bandgap of 1.831 eV on monolayer MoS_2_ grown on flat SiO_2_/Si, which accounts for the direct gap transition. At room temperature, the pristine monolayer MoS_2_ shows a high-quality strong PL peak associated with the band-to-band optical transition at the K point, as reported in previous studies [31,34]. The PL spectrum of MoS_2_ on the patterned substrate is located at 1.843 eV with a slightly low intensity compared to the one on flat SiO_2_/Si; however, these changes are possibly due to the local strain and minimal thickness variations at the trenches of the acquired region. Furthermore, we obtained the PL spectrum of as-grown three-layered MoS_2_ on TaN recording an optical bandgap of 1.839 eV, still measurable though appearing as a low-intensity peak when plotted together with the PL response from the other two substrates. The intensity reduction is possibly related to the different number of layers, being the PL peak intensity highly enhanced in the monolayer limit. When reduced at the single layer, the band gap in MoS_2_ shifts from indirect to direct [34]. The direct optical transitions happen between the conduction band minimum and the two-valence band maxima at the K point of the Brillouin zone, which splits due to the spin–orbit coupling. Here the acquired experimental bandgap values show strong emission claiming for a good quality monolayer MoS_2_, with reduced defect density, both on flat and pre-patterned SiO_2_/Si substrates, with peak values for A exciton (1.831 eV and 1.843 eV, respectively) well in agreement with values reported in literature [31].

**Figure 4:**
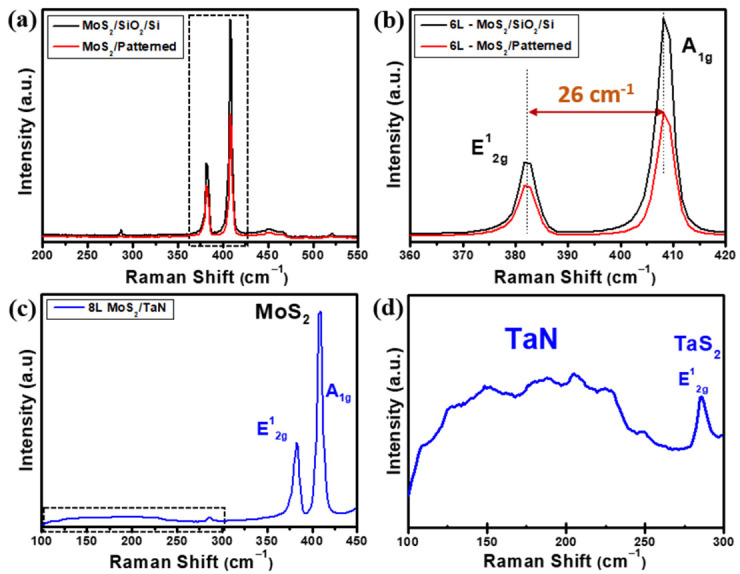
(**a**) Raman spectra obtained on vertically aligned MoS_2_ on pre-patterned (red) and equivalent thick flat (black) SiO_2_/Si substrate, (**b**) magnified Raman spectra of panel (**a**) evidencing E^1^_2g_ (in-plane), A_1g_ (out of plane) phonon modes of MoS_2_ and their frequency difference. (**c**) Raman spectrum of vertical aligned MoS_2_ on TaN substrate and (**d**) magnification of Raman spectrum in panel (**c**) in the low Raman shift region (dashed box in (**c**)), where the presence of co-deposited TaS_2_ peak is evidenced by its E^1^_2g_ (in-plane) phonon mode.

With a similar analytical approach as used for the flat MoS_2_ nanosheets, we considered micro-Raman spectroscopy to analyse the vertically aligned MoS_2_ growths. Figure 4a–d shows the Raman spectra of as-grown vertically aligned MoS_2_ flakes on flat SiO_2_/Si, pre-patterned SiO_2_/Si and TaN substrate, respectively. As shown in Figure 4a, the Raman spectrum obtained from MoS_2_ grown on pre-patterned SiO_2_/Si substrate (red line) clearly show the main two MoS_2_ phonon modes E^1^_2g_ and A_1g_ located at 382.9 cm^−1^ and 409.1 cm^−1,^ respectively. As a further assessment, a dedicated MoS_2_ growth on flat SiO_2_/Si substrate targeting similar MoS_2_ thickness as in the vertical growth case was performed, and its Raman spectrum is shown in Figure 4a (black line) for comparison. The frequency difference of the E^1^_2g_ and A_1g_ Raman modes gives 26 cm^−1^, as evidenced in Figure 4b, where the phonon mode region corresponding to the dashed box drawn in Figure 4a is plotted. Such value is compatible with the presence of more than 6 MoS_2_ layers and an overall thickness of >4.2 nm, considering the thickness of a MoS_2_ monolayer equal to 0.7 nm, as known from the literature [35,36]. Raman spectra of MoS_2_ on TaN shows MoS_2_ phonon modes at 381.6 cm^−1^ for in-plane E^1^_2g_ and 409.8 cm^−1^ for out-of-plane A_1g_ with a frequency difference of 28.2 cm^−1^, which corresponds to an eight layer-thick MoS_2_ on average. In addition, a broad range (100–250 cm^−1^) of Raman peaks (highlighted by the dashed box) is evident, confirming the presence of preserved TaN after MoS_2_ growth, as shown in Figure 4c. Figure 4d shows the enlarged Raman region limited by the dashed square in Figure 4c to evidence the presence of a weak Raman peak around 280 cm^−1^ assigned to the E_2g_ in-plane vibrational mode of 2H-TaS_2_, which is an indication of the reaction between the interface of TaN and excess sulfur during the CVD growth process. The evidence of 2H-TaS_2_ is unexpected based on thermochemistry considerations because the chemical reaction from Ta and S to form TaS_2_ happens at around 900 °C at ambient pressure. Additional factors, such as a supersaturated sulfur atmosphere, out-of-equilibrium conditions, chemistry kinetics or catalytic effects, should be further considered to fully understand the formation of TaS_2_. As noted above, the weak peaks on the broad region at low Raman shifts are assigned to TaN when stoichiometry modifications occur, either excess of Ta or excess N [32,33].

**Figure 5:**
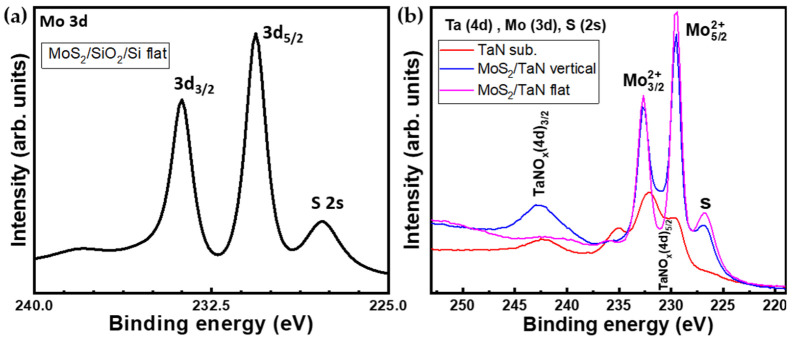
(**a**) XPS spectrum shows Mo(3d) and S(2p) core level regions of as-grown MoS_2_ on SiO_2_/Si (**b**) XPS spectra of Ta(4d), Mo(3d) and S(2s) core level regions of as-grown vertical (blue) and flat (magenta) MoS_2_ on TaN and bare TaN substrate (red).

XPS analysis was performed to investigate the elemental composition and chemical bonding of the as-grown MoS_2_. Figure 5a represents the XPS spectrum of the Mo(3d) and S(2s) spectral region for the CVD grown MoS_2_ monolayers. The spectral region contains the Mo 3d core-level line with Mo 3d 5/2 at 230.6 eV and Mo 3d 3/2 at 233.7 eV peaks, and the S 2s core-level line peaked at 227.8 eV, all pointing out to Mo-S bonding. The Mo peak positions are indicative of a MoS_2_ arranged in a majority trigonal prismatic 2H-phase, in agreement with Raman spectroscopy. These results are consistent with previous works on peak positions for MoS_2_ crystals. This observation constitutes the spectroscopic proof of the presence of Mo and S elements in a MoS_2_ compound. Figure 5b shows the XPS spectra recorded on vertical and flat aligned MoS_2_ growth (in blue and magenta, respectively) on TaN substrate together with the bare TaN substrate (red). The spectral windows correspond to Ta-4d, Mo-3d, and S-2s core-level lines. The XPS investigation reveals the presence of Mo, S and TaN in the MoS_2_ on TaN grown case. The TaN bare substrate (red line) XPS spectrum shows peaks at binding energies around 230.4 eV and 242.5 eV, which are distinctive of the 4d 5/2 and 4d 3/2 states of partially oxidized TaN, such as TaNO_x_ [37]. Such analysis underlies that the pristine TaN substrate is partially oxidized at the surface, possibly due to exposure to the environment. This could be the evidence for the persistence of the TaN layer after the MoS_2_ growth. XPS of MoS_2_ on TaN shows prominent peaks at binding energies of around 229.4 eV and 232.6 eV, which are assigned to the doublet of Mo 3d 5/2 and Mo 3d 3/2, respectively. In addition, the sulfur peak (S-2s), located at 226.7 eV, is seen in both flat and vertical MoS_2_. Interestingly, in the case of flat MoS_2_ on TaN we do not observe any TaN peak (magenta line) around binding energy 242.5 eV of TaN(O_x_), indicating that MoS_2_ nanosheets are fully covering the TaN film. However, in the vertically-grown MoS_2_ case (blue line), we clearly found an intense peak of TaNO_x_ at a binding energy of 242.7 eV (4d 3/2) and at 230.6 eV (4d 5/2), where an increase in the valley minimum between Mo related peaks is visible. As reasonable, in the case of vertically aligned MoS_2,_ a partial exposure of TaN substrate persists through the MoS_2_ domains due to the different growth modes, which compromises the full surface coverage. By comparing the TaN (red) and MoS_2_ on TaN (blue and magenta), it is also confirmed that TaN keeps preserved during the MoS_2_ CVD growth process conditions. Thus, the XPS analysis provides further experimental support to show the formation of flat and vertically aligned MoS_2_ on top of TaN substrate.

## 4. Conclusions

Increasing the control of the deposition process is mandatory for developing desired MoS_2_ production with fine electrical, optical, and chemical properties. Therefore, we successfully demonstrated a facile and controllable method for the synthesis of large-area MoS_2_ monolayer to a few layers from MoO_3_ and S powder precursors using a two-zone furnace AP-CVD system. We showed direct growth of large-area MoS_2_ on different substrates such as flat and pre-patterned SiO_2_/Si and TaN, giving an overview of the growth method. This enabled us to display an extensive portfolio of possible configurations spanning from flat MoS_2_ nanosheets with an atomically controlled thickness on the macro-scale to vertical MoS_2_ flakes to micro and nano-patterned MoS_2_ by design, thereby offering a versatile materials background to identify a good MoS_2_ configuration for a given target application. The configurational variability by changing the precursor positions through furnace movement helped the reaction in favoring the growth orientation. Furthermore, the control of temperature in the MoO_3_ reaction zone allowed for the growth on the different types of substrates. By optimizing the growth conditions, high-quality horizontally or vertically aligned MoS_2_ nanosheets were successfully achieved. In a wider context, our results may be of help to stimulate further exploitation in similar growth techniques when applied to 2D materials and their derivatives. We also believe our proposed methodology has the capability for the fabrication of MoS_2_ based applications, providing a facile synthesizing of TMDs even at the industrial level for a plethora of potential applications.

## Data Availability

Data can be available upon request from the authors.

## Supplementary Materials

The following supporting information can be downloaded at: https://www.mdpi.com/article/10.3390/nano12060973/s1, Figure S1. SEM image of MoS_2_ CVD growth on flat SiO_2_/Si substrate with no use of PTAS molecules. SEM image shows MoS_2_ isolated domains small in size, less than 10 μm. A satisfactory surface coverage is not achieved, leaving a relevant region of exposed substrate surface with no MoS_2_ growth. Figure S2. Picture of SiO_2_/Si substrate before and after the growth of 1 – 2 layer MoS_2_ over 1 × 1 cm2 lateral area. Figure S3. SEM images of MoS2 horizontally grown on SiO_2_/Si substrate, following the edge of the deposited area. The mapped region shows continuous growth over an extended region of several mm. Figure S4. TEM image cross sectional view of monolayer MoS2 on patterned substrate. (a) TEM cross section image shows that MoS_2_ grows with the basal plane exactly oriented parallel to the SiO_2_ surface. Here, the MoS_2_ flake is perfectly following the trenches of the pattern, as represented by the dark black line between the substrate and fixture glue (light gray color). (b) The red region in (a) is shown at higher magnification to ensure the monolayer are following the trench without rupture. Figure S5. SEM image shows cross-sectional view of vertically aligned MoS_2_ domains on patterned substrate. Figure S6. (a) AFM topographic image of monolayer MoS_2_ nanosheet on flat SiO_2_/Si substrate. (b) Height profile of single layer MoS_2_ along the dashed line in (a) claiming for 0.7 nm step height. AFM analysis evidences the morphology of the CVD grown MoS_2_ monolayer to be uniformly flat with sharp edges.

## References

[1] Chhowalla M., Shin H.S., Eda G., Li L.-J., Loh K.P., Zhang H. (2013). The chemistry of two-dimensional layered transition metal dichalcogenide nanosheets. Nat. Chem..

[2] Manzeli S., Ovchinnikov D., Pasquier D., Yazyev O.V., Kis A. (2017). 2D transition metal dichalcogenides. Nat. Rev. Mater..

[3] Wang Q.H., Kalantar-Zadeh K., Kis A., Coleman J.N., Strano M.S. (2012). Electronics and optoelectronics of two-dimensional transition metal dichalcogenides. Nat. Nanotechnol..

[4] Ryou J., Kim Y.-S., Santosh K.C., Cho K. (2016). Monolayer MoS_2_ bandgap modulation by dielectric environments and tunable bandgap transistors. Sci. Rep..

[5] Zeng H., Cui X. (2015). An optical spectroscopic study on two-dimensional group-VI transition metal dichalcogenides. Chem. Soc. Rev..

[6] Jariwala D., Sangwan V.K., Lauhon L.J., Marks T.J., Hersam M.C. (2014). Emerging device applications for semiconducting two-dimensional transition metal dichalcogenides. ACS Nano.

[7] Wang K., Puretzky A.A., Hu Z., Srijanto B.R., Li X., Gupta N., Yu H., Tian M., Mahjouri-Samani M., Gao X. (2019). Strain tolerance of two-dimensional crystal growth on curved surfaces. Sci. Adv..

[8] Martella C., Mennucci C., Lamperti A., Cappelluti E., de Mongeot F.B., Molle A. (2018). Designer shape anisotropy on transition-metal-dichalcogenide nanosheets. Adv. Mater..

[9] Martella C., Ortolani L., Cianci E., Lamperti A., Morandi V., Molle A. (2019). Large-area patterning of substrate-conformal MoS_2_ nano-trenches. Nano Res..

[10] Hus S.M., Ge R., Chen P.-A., Liang L., Donnelly G.E., Ko W., Huang F., Chiang M.-H., Li A.-P., Akinwande D. (2020). Observation of single-defect memristor in an MoS_2_ atomic sheet. Nat. Nanotechnol..

[11] Lo C.-L., Zhang K., Smith R.S., Shah K., Robinson J.A., Chen Z. (2018). Large-area, single-layer molybdenum disulfide synthesized at BEOL compatible temperature as Cu diffusion barrier. IEEE Electron. Device Lett..

[12] Lo C., Catalano M., Khosravi A., Ge W., Ji Y., Zemlyanov D.Y., Wang L., Addou R., Liu Y., Wallace R. (2019). Enhancing interconnect reliability and performance by converting tantalum to 2D layered tantalum sulfide at low temperature. Adv. Mater..

[13] Li G., Chen Z., Li Y., Zhang D., Yang W., Liu Y., Cao L. (2020). Engineering substrate interaction to improve hydrogen evolution catalysis of monolayer MoS_2_ films beyond Pt. ACS Nano.

[14] Voiry D., Fullon R.R., Yang J., de Carvalho Castro e Silva C., Kappera R., Bozkurt I., Kaplan D., Lagos M.J., Batson P.E., Gupta G. (2016). The role of electronic coupling between substrate and 2D MoS_2_ nanosheets in electrocatalytic production of hydrogen. Nat. Mater..

[15] Ozden A., Ay F., Sevik C., Perkgöz N.K. (2017). CVD growth of monolayer MoS_2_: Role of growth zone configuration and precursors ratio. Jpn. J. Appl. Phys..

[16] Fan X., Xu P., Li Y.C., Zhou D., Sun Y., Nguyen M.A.T., Terrones M., Mallouk T.E. (2016). Controlled exfoliation of MoS_2_ crystals into trilayer nanosheets. J. Am. Chem. Soc..

[17] Fabbri F., Rotunno E., Cinquanta E., Campi D., Bonnini E., Kaplan D., Lazzarini L., Bernasconi M., Ferrari C., Longo M. (2016). Novel near-infrared emission from crystal defects in MoS_2_ multilayer flakes. Nat. Commun..

[18] Tumino F., Grazianetti C., Martella C., Ruggeri M., Russo V., Li Bassi A., Molle A., Casari C.S. (2021). Hydrophilic character of single-layer MoS_2_ grown on Ag(111). J. Phys. Chem. C.

[19] Bhatnagar M., Gardella M., Giordano M.C., Chowdhury D., Mennucci C., Mazzanti A., Della Valle G., Martella C., Tummala P., Lamperti A. (2021). Broadband and tunable light harvesting in nanorippled MoS_2_ ultrathin films. ACS Appl. Mater. Interfaces.

[20] Bhatnagar M., Giordano M.C., Mennucci C., Chowdhury D., Mazzanti A., Della Valle G., Martella C., Tummala P., Lamperti A., Molle A. (2020). Ultra-broadband photon harvesting in large-area few-layer MoS_2_ nanostripe gratings. Nanoscale.

[21] Fan X., Xu P., Zhou D., Sun Y., Li Y.C., Nguyen M.A.T., Terrones M., Mallouk T.E. (2015). Fast and efficient preparation of exfoliated 2H MoS_2_ nanosheets by sonication-assisted lithium intercalation and infrared laser-induced 1T to 2H phase reversion. Nano Lett..

[22] Zhang F., Momeni K., Abu AlSaud M., Azizi A., Hainey M.F., Redwing J.M., Chen L.-Q., Alem N. (2017). Controlled synthesis of 2D transition metal dichalcogenides: From vertical to planar MoS_2_. 2D Mater..

[23] Miao C., Zheng C., Liang O., Xie Y.-H. (2011). Chemical Vapor Deposition of Graphene. Physics and Applications of Graphene-Theory.

[24] Tummala P., Lamperti A., Alia M., Kozma E., Nobili L.G., Molle A. (2020). Application-oriented growth of a molybdenum disulfide (MoS_2_) single layer by means of parametrically optimized chemical vapor deposition. Materials.

[25] Zhu D., Shu H., Jiang F., Lv D., Asokan V., Omar O., Yuan J., Zhang Z., Jin C. (2017). Capture the growth kinetics of CVD growth of two-dimensional MoS_2_. npj 2D Mater. Appl..

[26] Dos Santos R.B., Rivelino R., de Brito Mota F., Gueorguiev G.K., Kakanakova-Georgieva A. (2015). Dopant species with Al–Si and N–Si bonding in the MOCVD of AlN implementing trimethylaluminum, ammonia and silane. J. Phys. D Appl. Phys..

[27] Kakanakova-Georgieva A., Gueorguiev G.K., Yakimova R., Janzen E. (2004). Effect of impurity incorporation on crystallization in AlN sublimation epitaxy. J. Appl. Phys..

[28] Li H., Zhang X.H., Tang Z.K. (2019). Catalytic growth of large area monolayer molybdenum disulfide film by chemical vapor deposition. Thin Solid Film..

[29] Momeni K., Ji Y., Zhang K., Robinson J.A., Chen L.-Q. (2018). Multiscale framework for simulation-guided growth of 2D materials. Npj 2D Mater. Appl..

[30] Navarro-Moratalla E., Island J.O., Mañas-Valero S., Pinilla-Cienfuegos E., Castellanos-Gomez A., Quereda J., Rubio-Bollinger G., Chirolli L., Silva-Guillén J.A., Agraït N. (2016). Enhanced superconductivity in atomically thin TaS_2_. Nat. Commun..

[31] Martella C., Kozma E., Tummala P.P., Ricci S., Patel K.A., Andicsovà-Eckstein A., Bertini F., Scavia G., Sordan R., Nobili L.G. (2020). Changing the electronic polarizability of monolayer MoS_2_ by perylene-based seeding promoters. Adv. Mater. Interfaces.

[32] Stoehr M., Shin C.-S., Petrov I., Greene J.E. (2007). Raman scattering from epitaxial TaNx(0.94 ≤ x ≤ 1.37) layers grown on MgO(001). J. Appl. Phys..

[33] Huang J., Wang X., Hogan N.L., Wu S., Lu P., Fan Z., Dai Y., Zeng B., Starko-Bowes R., Jian J. (2018). Nanoscale artificial plasmonic lattice in self-assembled vertically aligned nitride–metal hybrid metamaterials. Adv. Sci..

[34] Splendiani A., Sun L., Zhang Y., Li T., Kim J., Chim C.-Y., Galli G., Wang F. (2010). Emerging photoluminescence in monolayer MoS_2_. Nano Lett..

[35] Chakraborty B., Matte H.S.S.R., Sood A.K., Rao C.N.R. (2013). Layer-dependent resonant Raman scattering of a few layer MoS_2_. J. Raman Spectrosc..

[36] Lee Y.-H., Yu L., Wang H., Fang W., Ling X., Shi Y., Lin C.-T., Huang J.-K., Chang M.-T., Chang C.-S. (2013). Synthesis and transfer of single-layer transition metal disulfides on diverse surfaces. Nano Lett..

[37] Husain S., Akansel S., Kumar A., Svedlindh P., Chaudhary S. (2016). Growth of Co_2_FeAl Heusler alloy thin films on Si(100) having very small Gilbert damping by Ion beam sputtering. Sci. Rep..

