# Supplementary material for: Ambient Pressure Chemical Vapor Deposition of Flat and Vertically Aligned MoS2 Nanosheets
Source: Nanomaterials (Basel). 2022 Mar 16;12(6):973. doi: 10.3390/nano12060973 (PMC8949030; doi:10.3390/nano12060973)
Supplement: Supplementary file 1 [file nanomaterials-12-00973-s001.zip › nanomaterials-1592689-supplementary.pdf]

nanomaterials

Supplementary Materials

Ambient Pressure Chemical Vapor Deposition of Flat and Vertically Aligned MoS_2 Nanosheets

Pinaka Pani Tummala [1,2,3], Christian Martella [1], Alessandro Molle [1] and Alessio Lamperti [1,*]

[1] Institute for Microelectronics and Microsystems (CNR-IMM), Unit of Agrate Brianza, via C. Olivetti 2, I-20864 Agrate Brianza, Italy; pinakapani.tummala@mdm.imm.cnr.it (P.P.T.); christian.martella@mdm.imm.cnr.it (C.M.); alessandro.molle@mdm.imm.cnr.it (A.M.)
[2] Dipartimento di Matematica e Fisica, Università Cattolica del Sacro Cuore, via della Garzetta 48, 25133 Brescia, Italy
[3] Department of Physics and Astronomy, Katholieke Universiteit Leuven (KU Leuven), Celestijnenlaan 200D, 3001 Leuven, Belgium
* Correspondence: alessio.lamperti@mdm.imm.cnr.it (A.L.)

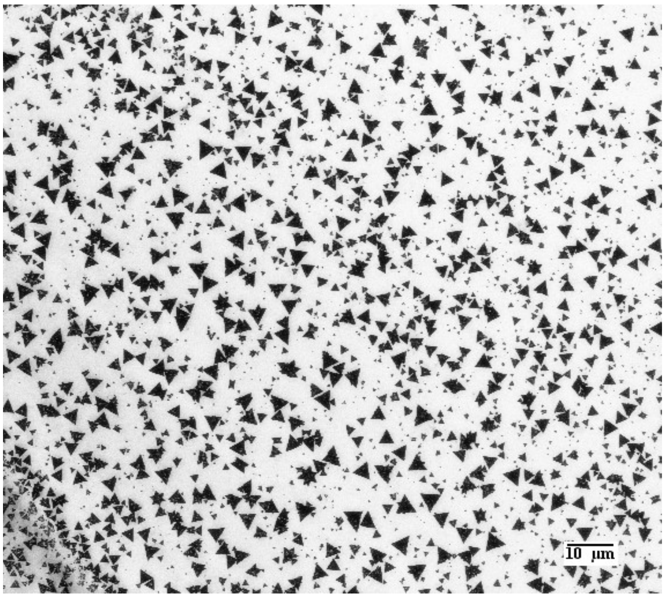

Figure S1. SEM image of MoS_2 CVD growth on flat SiO_2/Si substrate with no use of PTAS molecules. SEM image shows MoS_2 isolated domains small in size, less than 10 μm. A satisfactory surface coverage is not achieved, leaving a relevant region of exposed substrate surface with no MoS_2 growth.

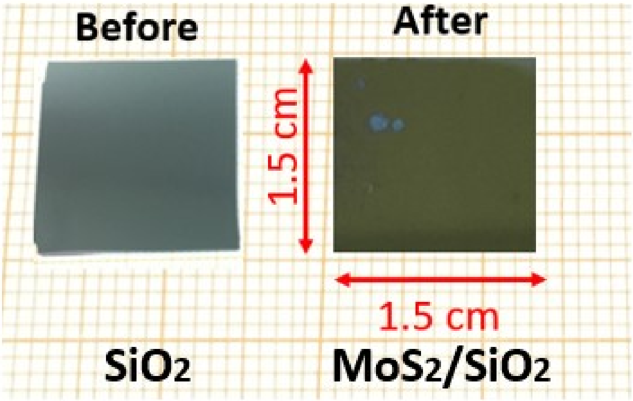

Figure S2. Picture of SiO_2/Si substrate before and after the growth of 1 – 2 layer MoS_2 over 1 x 1 cm^2 lateral area.

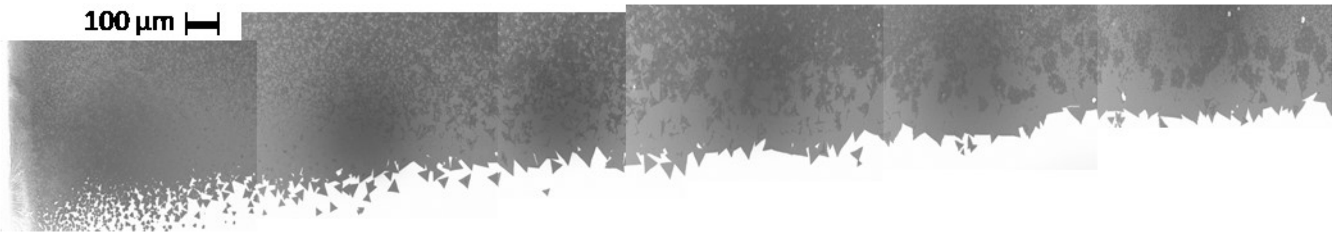

Figure S3. SEM images of MoS_2 horizontally grown on SiO_2/Si substrate, following the edge of the deposited area. The mapped region shows continuous growth over an extended region of several mm.

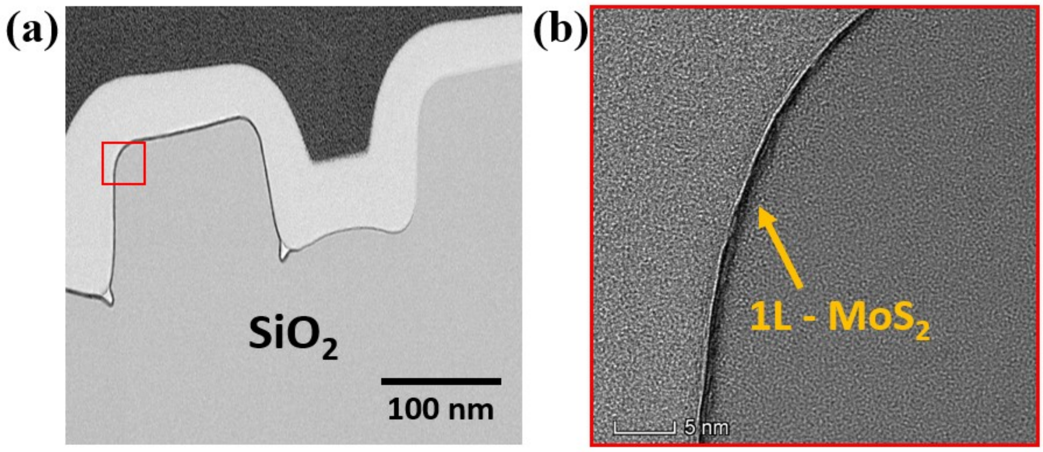

Figure S4. TEM image cross sectional view of monolayer MoS_2 on patterned substrate. (a) TEM cross section image shows that MoS_2 grows with the basal plane exactly oriented parallel to the SiO_2 surface. Here, the MoS_2 flake is perfectly following the trenches of the pattern, as represented by the dark black line between the substrate and fixture glue (light gray color). (b) The red region in (a) is shown at higher magnification to ensure the monolayer are following the trench without rupture.

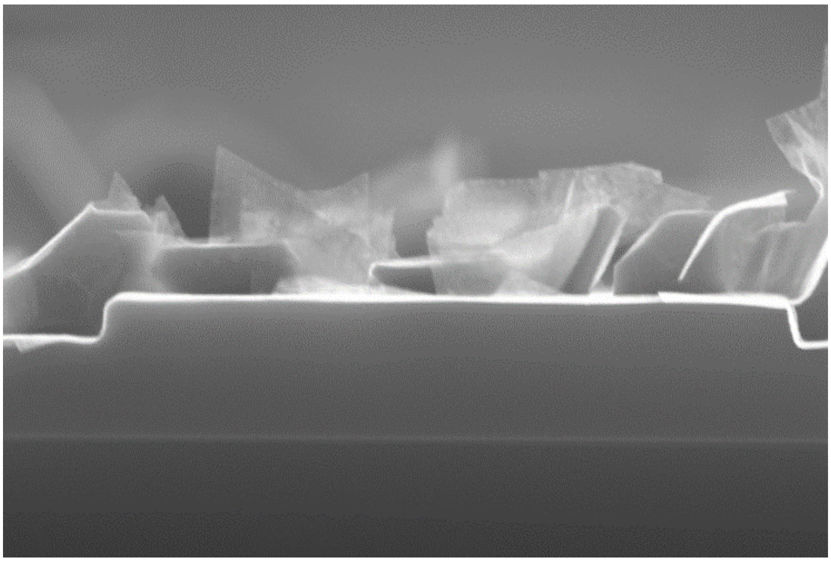

Figure S5. SEM image shows cross-sectional view of vertically aligned MoS_2 domains on patterned substrate.

SEM cross sectional image confirms the vertically aligned triangular MoS_2 domains on patterned substrate is consistent with the homogeneity over the patterned substrate as shown in **Figure S5**.

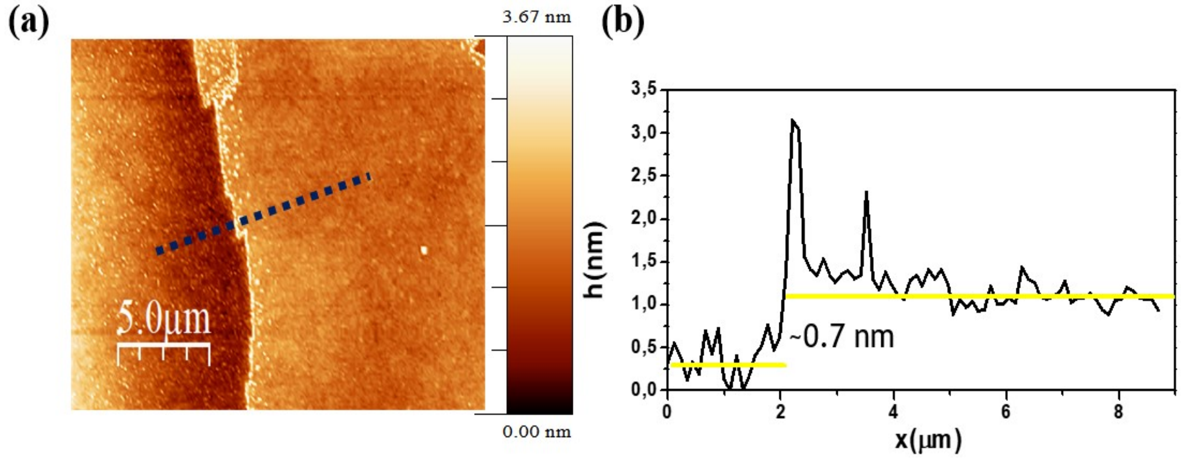

Figure S6. (a) AFM topographic image of monolayer MoS_2 nanosheet on flat SiO_2/Si substrate. (b) Height profile of single layer MoS_2 along the dashed line in (a) claiming for 0.7 nm step height. AFM analysis evidences the morphology of the CVD grown MoS_2 monolayer to be uniformly flat with sharp edges.
